# The Care of New York Children’s Teeth

**Published:** 1910-05-15

**Authors:** Herbert L. Wheeler

**Affiliations:** New York, N. Y.


### THE CARE OF NEW YORK CHILDREN'S TEETH.

BY DR. HERBERT L. WHEELER, NEW YORK, N. Y.

The fact that the National Dental Association has taken up the question of dental hygiene is a most encouraging sign. They are beginning to take the proper position, starting at the fundamentals. The experience that we have had in New York leads me to believe that there are not enough dentists in America to attend to the carious teeth in the city of New York alone, and unless we arrive at the point where it will be possible to prevent some of this trouble the situation is hopeless. The only way of successfully coping with the situation, as has been said here to-night, is to educate the people to the necessity of having a clean mouth if they would have a healthy mouth, although I am quite sure that the present knowledge of what causes the destruction of the human teeth is not sufficient for any dentist to say that it is possible to have in every mouth absolutely clean teeth, which I do not believe. In practicing exclusively among people who are able to pay dental bills without regard to expense, and who take all the care possible of their mouths and teeth, I find that the children, although surrounded by every necessary appliance and by servants to insist upon proper oral care, exhibit a great deal of caries. The filling of these carious teeth takes a great deal of time. The placing in the books of the schools rules and regulations in regard to the care of the teeth does not sufficently impress the student or the teacher. It is a well-known fact that at the present time every state has laws requiring that hygiene be taught for a certain number of hours each week, but no text-book is so much neglected as are the books on hygiene.

In New York city, where I took the pains to find out the actual conditions, the board of health and the department of hygiene are attempting by actual comparative methods to find out whether with clean mouths the children show better health and better mental conditions. These experiments are being carried out in the following way: A certain number of children from some of the schools are sent to the dental clinics, and their mouths are and will be kept in order for four or five years; others living in the same community under the same conditions, but without dental care, will be kept under observation for the same length of time, and at the end of that time we shall be able to decide whether those who have received the attention of the dentist, the nose specialist, and the ear specialist are in better condition than those who have not received this attention. Then we shall be able to speak of facts. At the present time the facts are not known, as statements made here have shown.

A most remarkable condition was shown by the examinaion of 172,000 school children in New York, namely, that ninety-five per cent. needed dental care, and that seventy-five per cent. have never been in a dental office. Dental clinics have been established in the Bellevue Hospital, in three other hospitals, and in several of the schools, and furnished with the best equipment; the city is bearing the expense and would establish many more clinics if it were possible to secure dentists to attend those clinics. A most remarkable fact in connection with this work of establishing dental clinics in New York city is that not one of them has been brought about by the dentists. Personally, I have struggled for three years to induce the dentists to take up this work, but it has only been done in a desultory way. What has been accomplished in New York has been done by charitable organizations and through men who have been interested in social settlements and in the physical development and welfare of the people, and the education carried on in this way has aroused the public to such an extent that they are beginning to criticize the dentists, many times unjustly of course, because he has to be educated as the public is educated. The dentist must therefore take heed of this work unless he wishes to pay the penalty of his neglect, and it is my opinion that unless the local organizations take up this work in a practical way, work on an intelligent basis, and bring their results before the National Association, time, energy and money will be wasted. The National Association cannot carry on this work without having obtained practical facts by practical experience. The Dental Hygiene Council of Massachusetts has accomplished some work in oral hygiene, distributing circulars similar to those issued by the National Committee. Many of the men in Massachusetts and in some parts of Connecticut are working in harmony with the school committees, with the charity organizations, and with public institutions such as boards of health, and I assure you it is up-hill work. All that has been accomplished is the examination of the mouths of the children of a few schools in Boston, carried on under Dr. Potter, who found that in Brookline, the wealthiest town in America, seventy per cent. of the school children need dental service. Just think what the condition must be in New York, where so many of the people are too poor to buy sufficient clothing! Just think under what difficulties we shall have to labor, and what an enormous amount of labor is involved, if we are to accomplish anything for these children. In one of the clinics in New York the work was taken up by the dentists last October, and has been carried on to the present time, but two dentists working every afternoon five times a week have only succeeded in completing the work in a few hundred children. Look at what we have to face, if we are to do anything for these 172,000 children that have been examined, who represent hardly a quarter of the public school children that need attention in New York city. I merely bring this to your attention to show both sides of the question.

Agein let me emphasize that we must get to work on this question in a practical way. Let us first demonstrate how much time will be necessary to carry on this reparative work, and then we must set about to find some way of carrying on preventive dentistry rather than reparative dentistry, and we must educate the public to assist us through their organizations, or else we can do practically nothing.—*Cosmos.*

